# Measuring qualities needed for interdisciplinary work: The Intellectual Virtues for Interdisciplinary Research Scale (IVIRS)

**DOI:** 10.1371/journal.pone.0312938

**Published:** 2024-11-15

**Authors:** Claudia E. Vanney, Belén Mesurado, J. Ignacio Aguinalde Sáenz

**Affiliations:** 1 Instituto de Filosofía, Universidad Austral, Buenos Aires, Argentina; 2 CONICET–Consejo Nacional de Investigaciones Científicas y Técnicas, Buenos Aires, Argentina; 3 Facultad de Ciencias Biomédicas, Universidad Austral, Buenos Aires, Argentina; ECHS: Universidade de Tras-os-Montes e Alto Douro Escola de Ciencias Humanas e Sociais, PORTUGAL

## Abstract

Previous work has suggested that the problems hindering the success of interdisciplinarity could be overcome by fostering certain intellectual character strengths in scholars. However, how to assess and cultivate the specific virtues required for interdisciplinarity among researchers is still a matter of inquiry. The general objective of this paper was to develop a psychometric instrument to assess intellectual virtues that are essential for interdisciplinary inquiry among researchers. To achieve this goal, two studies were conducted. Study 1 developed a new scale and studied its correlation with other validated measures. Study 2 focused on conducting a confirmatory analysis of the structure obtained in Study 1 and investigated the relationships between the new scale and the researchers’ levels of (i) experience and productivity in interdisciplinary collaboration and (ii) satisfaction regarding the results of their interdisciplinary inquiry. The EFA conducted for Study 1 identified four dimensions: (1) intellectual empathy, (2) open-mindedness and intellectual humility, (3) intellectual perseverance, and (4) curiosity. Indeed, the pools of items that were initially developed to measure intellectual humility and open-mindedness in a separate way converged into a unique factor or dimension. The confirmatory factor analysis conducted for Study 2 corroborated the four-dimensional structure observed in Study 1 via a new different sample. In addition, Studies 1 and 2 also analyzed convergent validity through the AVE and correlated the IVIRS with other scales that measure intellectual virtues (open-mindedness, curiosity, intellectual humility, and perseverance) in a general epistemic context. The second study demonstrated that researchers with significant experience, productivity, and satisfaction in the context of interdisciplinary investigation also presented elevated levels of the intellectual virtues that we identified as essential for such research. Our analysis demonstrates that the IVIRS is a valid measure of the intellectual virtues needed for interdisciplinarity and paves the way for the future design of interventions to cultivate these character traits in scholars.

## Introduction

In many academic areas, interdisciplinary research has become a desired goal. It is a practice that produces new knowledge by integrating insights from different fields. It involves establishing connections between various branches of knowledge, fostering the emergence of new ideas, gaining enrichment from diverse perspectives, and promoting greater collegiality and academic collaboration. However, many challenges are associated with interdisciplinarity [1] because this kind of approach requires complex social and intellectual processes as a team-based type of research [2–4]. Indeed, social coordination among researchers of different disciplines can be difficult due to the varying levels of expertise and understanding of each other’s work [5]. In addition, there is a need to possess sufficient knowledge in several domains to be able to collaborate with experts from other fields, which implies considerable time commitment. Moreover, some researchers have noted that interdisciplinary research is more likely to be superficial [6]. In short, interdisciplinary success requires diligent translation, a shared understanding, and respect for the slow growth of specialized knowledge [7], all of which are challenging requirements.

Although empirical studies on interdisciplinary cognition at the individual level have largely been neglected in the literature [8], recent investigations have suggested that cultivating specific epistemic virtues among researchers can help overcome the barriers to interdisciplinary success [9]. Like any other researcher, an interdisciplinary scholar must possess a wide range of intellectual skills and capabilities. However, because interdisciplinarity involves extending the use of intellectual character strengths beyond one’s own field and reflecting deeply on areas other than one’s own, deploying epistemic virtues within the framework of interdisciplinary work is also more demanding. Additionally, interdisciplinary researchers must cultivate specific dispositions that enable them to think collaboratively.

In this context, the area of philosophy known as virtue epistemology presents a promising strategy for studying the dispositions of researchers and addressing the problems we have mentioned. Since the pioneering works of Ernest Sosa and Linda Zagzebski [10, 11], virtue epistemology has been developed as a novel approach for solving certain challenges within the philosophy of knowledge [12, 13]. The common thesis of these authors was to shift the focus of epistemology from the evaluation of properties of beliefs to the evaluation of certain properties or dispositions of persons [14]. Hence, the distinctive feature of the approach proposed by virtue epistemology lies in emphasizing the consideration of cognitive faculties and the virtues that perfect them to account for the possibility of attaining knowledge of truth [15]. However, virtue epistemology is a research program that includes a diverse collection of approaches to epistemology [13].

Some virtue epistemologists consider intellectual virtues as the proper character traits for excellent thinkers [11]. Examples of these virtues are curiosity, intellectual humility, open-mindedness, and intellectual perseverance, among those that have received the most attention. In both philosophy and psychology, numerous studies have focused on applying intellectual virtues to different fields, such as education [16] and scientific research [17]. Another focus of interest has been the relationship between virtue epistemology and the philosophy of science [18]. Given that in this context, intellectual virtues are defined as personal qualities that support the pursuit of knowledge across domains, it would be logical to assume that they may, in turn, contribute to overcoming obstacles to interdisciplinary research [18].

Following rigorously the consensual qualitative analysis, a methodology that suggests a sample size of 8–15 participants [19], Vanney et al. [20] analyzed nine interviews involving researchers with a long personal trajectory of interdisciplinary research in the sciences and the humanities to identify intellectual virtues relevant to interdisciplinary research. Three domains were identified in this research: intellectual virtues, social virtues, and interpersonal intellectual virtues. In addition, six categories of intellectual virtues were highlighted: intellectual humility, open-mindedness, intellectual perseverance, curiosity, intellectual honesty, and intellectual autonomy. Five categories within the domain of social virtues were emphasized: friendship, kindness, collaborative spirit, sense of humor, and communication skills. Finally, four categories within the domain of interpersonal intellectual virtues were identified: intellectual empathy, intellectual respect, intellectual trust, and intellectual generosity. Additionally, the authors ranked the frequency of the categories in terms of the number of respondents who mentioned them. Only four virtues were mentioned by all the scholars: intellectual humility, open-mindedness, intellectual perseverance, and intellectual empathy. Thus, according to this study, these virtues represent interdisciplinary researchers’ most salient character traits.

On the basis of these findings, this paper hypothesizes that an interdisciplinary researcher needs several virtues to conduct interdisciplinary research successfully. As a starting point, we propose a multidimensional instrument that includes the four critical virtues for interdisciplinary work previously mentioned while also incorporating curiosity. Although curiosity was not universally mentioned in the study just discussed, seven of the nine interviewees highlighted that it is an intrinsic motivator for any epistemic endeavor.

### The conceptualization of these virtues in an interdisciplinary research context

#### Intellectual humility

Researchers’ focus on intellectual humility has increased over the past decade, resulting in numerous works on this topic [21, 22]. Among its various conceptualizations, recognizing one’s own intellectual limitations [23–26] and, more comprehensively, embracing one’s own intellectual strengths and weaknesses [27] stand out. When performing scholarly work in a group context, as in the case of interdisciplinary research, intellectual humility also implies an awareness of the strengths and weaknesses of one’s own field. In this sense, this virtue can be embodied when one recognizes that other disciplines always have something new to teach us and when one is willing to admit that one’s opinions are limited in the context of interdisciplinary discussions. Intellectual humility is often characterized by an individual’s readiness to correct and learn from his or her mistakes.

#### Open-mindedness

Open-minded individuals are willing to consider the merits of alternative viewpoints, even if those viewpoints differ from their own, because they believe that doing so can help them discover the truth [28–30]. People can transcend their biases and become more receptive to new ideas and knowledge by being open-minded. Open-mindedness can be viewed as the willingness to learn new things, consider different theoretical alternatives, and accept other views concerning an issue if they are well founded [31]. Interdisciplinary research evidently requires open-mindedness, which involves actively listening to and considering perspectives drawn from other disciplines. This quality also entails being willing to change one’s opinion when presented with well-founded arguments by colleagues from other fields. In interdisciplinary work, this virtue is also embodied when the individual desires to approach research topics from multiple disciplinary perspectives.

#### Intellectual perseverance

Virtue epistemologists usually emphasize that epistemic goals are challenging and require continuous effort over time. Intellectual perseverance is necessary to persist in one’s intellectual endeavors despite obstacles [32, 33]. Angela Duckworth made significant contributions to our understanding of this notion in psychology by studying grit and developing a scale to measure this factor [34–36]. As intellectual perseverance is essential for success in any academic endeavor, it is crucial in interdisciplinary research because it enables team members to overcome the difficulties associated with interdisciplinarity, thus helping them maintain their intellectual efforts over time.

#### Curiosity

This intellectual virtue encourages the individual to investigate and inquire about topics that exceed his or her understanding without accepting simplistic or facile solutions [37]. As an epistemic virtuous quality, curiosity involves being appropriately discerning (by being aimed at the correct subject), timely (by emerging in the appropriate circumstances), and exacting (by avoiding becoming contented too easily) [38]. Curiosity is an intrinsic motivator that sparks and sustains the learning process [16]. Furthermore, curiosity plays a vital role in successful epistemic collaboration [39]. In interdisciplinary research, this virtue is characterized by a strong desire to learn and achieve a more comprehensive and profound understanding with the help of disciplines other than one’s own.

#### Intellectual empathy

A recent paper identified a subgroup of intellectual virtues known as interpersonal intellectual virtues [40]. These virtues are “defined as personal character traits that facilitate the reciprocal acquisition and distribution of knowledge *with* and *through* other people” (p. 173). They aim for the epistemic good like all intellectual virtues, but they apply interpersonally, like social virtues. Intellectual empathy is a paradigmatic example of an interpersonal intellectual virtue. This virtue entails two ways of understanding another person’s thinking: (i) seeing things from their point of view and (ii) feeling moved to follow the other person’s cognitive process. In interdisciplinary research, intellectual empathy helps us understand *how* scholars from different disciplines approach a common problem with the aim of imitating that approach to complement their own methodology.

### Measuring intellectual virtues

Assessments of intellectual virtues are still in an incipient stage of development, with a few exceptions, such as intellectual humility. In fact, many scales have been proposed to measure intellectual humility, such as those advanced by Alfano [41], Leary et al. [42], and Krumrei-Mancuso [43], among others. Despite different scales being based on diverse categorizations of intellectual humility, they all generally consider intellectual humility to be a stable attribute of a person or personality [44]. An instrument that deserves special attention or consideration in this context is Leary’s 6-item unidimensional scale [42], which focuses on intellectual humility’s essential features rather than its consequences or behavioral aspects. Leary’s scale is noteworthy since it was developed on the basis of a theoretical consensus among leading virtue epistemology scholars and expert psychologists who study humility.

The scale developed by Leary et al. emphasizes the metacognitive core of intellectual humility, which involves recognizing the limits of one’s own knowledge and being aware of one’s own fallibility. Other multidimensional instruments have also incorporated into the construct some socio-behavioral aspects that can be considered distinct virtues, derived from and closely related to intellectual humility, such as modesty, the ability to remain cognitively open to counterarguments (open-mindedness), or respect for other people’s views (intellectual respect), among others. For a more complete comparison of the diverse definitions of intellectual humility and their cognitive emphasis in different scales, see the work of Porter et al. [22].

Recent research has called for studies on intellectual humility to move beyond assessing the trait in isolation and to explore how it interacts with other intellectual virtues to promote a good intellectual character [45]. While several scales have been developed to evaluate individual intellectual virtues, e.g., curiosity [46, 47], open-mindedness [48–50], and grit [35], very few studies have comprehensively assessed the notion of a virtuous intellectual character. On the basis of virtue-based self-assessments [16], a 23-item scale that aims to measure virtuous intellectual character (VICS) was recently proposed and validated in Argentina [51]. This instrument focuses on five dimensions of a virtuous intellectual character: (i) attentiveness, (ii) open-mindedness, (iii) curiosity, (iv) carefulness, and (v) intellectual autonomy. On the other hand, it should be noted that, at present, there are no scales specifically designed for measuring a virtuous intellectual character in relation to interdisciplinary work.

### Measures of interdisciplinarity

In 2005, the US National Academy of Sciences [52] operationally defined interdisciplinarity as “a mode of research by teams or individuals that integrates information, data, techniques, tools, perspectives, concepts, and/or theories from two or more disciplines or bodies of specialized knowledge to advance fundamental understanding or to solve problems whose solutions are beyond the scope of a single discipline or area of research practice" (p. 2).

Since then, scholarly interest in measuring the quality of interdisciplinarity has increased. The importance of quality indicators and proper assessment processes for interdisciplinary initiatives has been discussed [53]. Generic evaluation principles [54] and indicators of interdisciplinarity in documents pertaining to the practices employed by research funders and policy-makers have been proposed [55], and the experiences of interdisciplinary researchers have been collected for the purposes of research evaluation [56].

Furthermore, assessments have been developed to measure partial aspects of interdisciplinarity, such as the interdisciplinarity of publications based on bibliometric approaches [57–59], the interdisciplinarity of a research system [60], and the disciplinary diversity of interdisciplinary papers [61, 62]. More comprehensively, pathways for assessing interdisciplinary initiatives have also been presented recently on the basis of a systematic review [63].

Thus, numerous studies have proposed different metrics for evaluating interdisciplinary initiatives (e.g., papers, research proposals, and programs), including objective data regarding the researcher’s background (such as the researcher’s field of training, academic affiliation, coauthorships, and citations from other disciplines). However, very little research has focused on developing self-reported assessments of the character traits, expertise, or productivity exhibited by individual researchers in the context of interdisciplinary work. Although some research has focused on this topic, it has emphasized specific areas, such as biomedical sciences or engineering [64, 65].

On a topic more similar to the research on which this paper focuses, three scales for measuring aspects of the collaborative processes of a research center have been developed: (1) perceived satisfaction with collaboration, (2) trust and respect in a collaborative setting, and (3) the impact of collaboration on the research process. These scales were developed and validated by Masse et al. [66] based on a questionnaire administered to 216 researchers at the Transdisciplinary Tobacco Use Research Centers. Items from scales (1) and (2) measure various aspects of the character of interdisciplinary researchers, and in this sense, these scales could be viewed as antecedents of our study. Moreover, in this paper, we have used the scale (3) to evaluate the level of satisfaction regarding the results of interdisciplinary research.

In addition, the results of a survey involving 214 early-career researchers from interdisciplinary departments or enrolled in interdisciplinary projects revealed that both context and attitude play important roles in raising awareness, shaping abilities, and reporting interdisciplinary achievements [67]. This survey also supports the relevance of our paper’s goal of developing a more detailed and in-depth evaluation of the intellectual character traits exhibited by interdisciplinary researchers.

### General objectives of the paper

The general purpose of the investigation presented in this article is to develop a psychometric instrument for assessing the intellectual virtues of researchers that are essential for interdisciplinary inquiry. Accordingly, (i) the instrument is designed, (ii) its validity and reliability are examined, and (iii) the question of whether individuals with significant experience and productivity in interdisciplinary work exhibit high levels of the virtues assessed by the scale is investigated. Two studies were conducted to achieve this general objective.

The proposed quantitative tool is essential for evaluating future interventions that may be designed to cultivate the intellectual character necessary for interdisciplinary work among young researchers.

## Study 1: Scale development and correlations with other validated measures

The primary objective of Study 1 is to develop a scale for measuring the intellectual virtues associated with interdisciplinary research. On the basis of previous qualitative research conducted by Vanney et al. [20], we aim to create a scale that measures five essential intellectual virtues that are required for interdisciplinary research, namely, (1) intellectual humility, (2) open-mindedness, (3) intellectual perseverance, (4) curiosity, and (5) intellectual empathy. A second objective of this study is to evaluate the new scale’s content, construct, convergent, and discriminant validity, as well as its reliability.

The intellectual humility scale by Leary and colleagues [42], the curiosity and open-mindedness subscales of a virtuous intellectual character scale (VICS) by Mesurado and Vanney [51], and the perseverance subscale of the Grit Scale by Duckworth and colleagues [68] will be used to study the convergent validity of the new scale. Existing scales measure intellectual virtues in a general epistemic context, unlike the new scale, which aims to assess the intellectual virtues associated with interdisciplinary research.

### Method

#### Development of the items

First, a team of three researchers was formed, including an academic with a background in science and philosophy, particularly in virtue epistemology; a psychologist specializing in psychometrics; and an academic with dual degrees in philosophy and psychology.

Based on a thorough review of the virtue epistemology literature, particularly the description of the intellectual virtues required for interdisciplinary research presented in a recent qualitative study [20], six items were developed to evaluate each of three epistemic virtues (open-mindedness, perseverance, and curiosity) in the specific context of interdisciplinary research, and seven items were developed to assess each of the other two intellectual virtues (intellectual humility and intellectual empathy) in an interdisciplinary environment. The initial scale included a total of 32 items.

#### Content validity

Five researchers who had previous experience conducting interdisciplinary research between the humanities and the natural sciences were contacted to serve as judges. A demographic description of the judges can be found in Table 1. These judges were asked to assess (i) the relevance of the proposed items with respect to each virtue and (ii) the clarity with which the statements were formulated.

**Table 1:** Demographic description of the researchers who acted as judges for assessing the items.

Interviewees	Age	Sex	Degree	Ph.D.	Interdisciplinary experience in years	Country of academic activity	Number of interdisciplinary projects led or participated in
Judge 1	62	F	Engineering Philosophy	Ph.D. Philosophy	25	Argentina	15
Judge 2	76	M	Philosophy Theology	Ph.D. Philosophy	30	Argentina	20
Judge 3	38	M	Psychology Philosophy	Ph.D. Psychology	7	Chile	5
Judge 4	43	F	Education	Ph.D. Education	8	Argentina	9
Judge 5	49	M	Philosophy	Ph.D. Philosophy	10	Argentina	9

Regarding the relevance of the items, the results indicated 100% agreement concerning 26 items and 80% agreement regarding 5 items. Only one item received 40% agreement and was therefore eliminated from subsequent studies. The judges made suggestions concerning ways of clarifying or simplifying the wording of 10 of the items (30%). For example, an original item was: “When researching, I accept that criticism from colleagues in other areas can *enrich* my work,” and the judges suggested changing the verb to emphasize a humble attitude: “When researching, I accept that criticism from colleagues in other areas can *improve* my work.” These modifications were included in the version used for subsequent analyses.

#### Participants and procedure

Study 1 was conducted with a nonprobabilistic data sample. The authors of this article have directed several interdisciplinary research projects in the last 15 years, so they have 522 researchers from Latin America in their network. To these 522, they also added other scholars belonging to important interdisciplinary research groups from countries in the region obtained through the internet. Thus, to recruit participants, a list of 800 researchers from Latin America was drawn, whose areas of expertise broadly covered the entire spectrum of academic disciplines from the natural and social sciences to the humanities.

For Study 1, 400 contacts were emailed, asking them to complete an anonymous online survey and share it with other researchers, following a snowball methodology. The same procedure was used with the remaining 400 contacts for Study 2.

The project included an online questionnaire for university teachers and researchers. Since the participants were well educated adults, the online survey was anonymous and contained unidentifiable information, and it did not collect private information from the participants; thus, no IRB application was necessary. Written informed consent was obtained, and all procedures performed in the studies were in accordance with the 1964 Helsinki Declaration and its later amendments or comparable ethical standards. The recruitment period started on September 5, 2023, and ended on October 5, 2023.

In both studies, to determine the final sample, the inclusion criterion was being a professor at a Latin American university. The exclusion criteria were (i) not having completed university studies, (ii) not working at a university or research center, or (iii) doing research or teaching in countries outside Latin America.

The sample used for Study 1 included 318 academics between the ages of 24 and 81 years (mean age = 48.58, SD = 12.28); 52.5% of these academics were male, 47.2% were female, and 0.3% were nonbinary. In terms of the highest educational level attained by the participants, 14.7% had completed a university education, 25.5% had completed studies for a specialization or a master’s degree, 38.7% had completed doctoral studies, and 21.1% had completed postdoctoral studies. Additionally, 11.3% of the participants reported performing teaching work, 27.1% reported performing disciplinary research, 20.1% reported performing interdisciplinary research, and 41.5% reported performing both types of research (disciplinary and interdisciplinary).

Webpower online software was used to determine the sample size’s statistical power using the procedure suggested by Satorr & Saris [69]. The effect size was 0.1, the significance level (alpha) was 0.05, the sample size was 318, and the degree of freedom was 210, resulting in a sample size’s statistical power of .43. Moreover, a power analysis based on the RMSEA [70] was also performed via Webpower software. The RMSEA H_0_ was 0, the RMSEA H_1_ was 0.05, the significance level (alpha) was 0.05, the sample size was 318, and the degree of freedom was 210, resulting in a sample size of statistical power of 1.

#### Instruments

*Intellectual Virtues for Interdisciplinary Research Scale (IVIRS)*. This scale included 31 items at this stage because one item was discarded in accordance with the judges’ suggestion, as indicated above. This initial version of the instrument aimed to measure (i) intellectual humility (6 items, e.g., ‘When researching, I am willing to acknowledge mistakes based on evidence provided by colleagues from other disciplines’), (ii) open-mindedness (6 items, e.g., ‘When researching, I am willing to change what I think after an exchange with colleagues from other disciplines’), (iii) perseverance (6 items, e.g., ‘When working with people from other fields, I strive to reach the end goal even though interdisciplinary work is arduous’), (iv) curiosity (6 items, e.g., ‘When researching, I am curious to learn how things are explained in different disciplines’), and (v) intellectual empathy (7 items, e.g., ‘When working with people from other fields, I try to understand how they reach certain conclusions’). A 5-point Likert scale (ranging from ‘very much’ = 5 to ‘not at all’ = 1) is used to score each item.

*Intellectual Humility Scale*. Leary and colleagues [42] developed a 6-item scale to measure intellectual humility. This unidimensional instrument includes items such as ‘In the face of conflicting evidence, I am open to changing my opinions’. Each item is scored on a 5-point Likert scale ranging from ‘not at all like me’ = 1 to ‘very much like me’ = 5. The total score is obtained by taking the average of the scores for each item included in the scale. A high score indicates a high level of intellectual humility. The McDonald’s omega coefficient (ω) for the Intellectual Humility Scale was ω = .77.

*Virtuous Intellectual Character Scale (VICS)*. On the basis of virtue-based self-assessments [16], a 23-item scale validated by Mesurado and Vanney [51] was used to measure intellectual virtues in a general epistemic context. The VICS measures five intellectual virtues: (1) intellectual autonomy, (2) attentiveness, (3) carefulness, (4) curiosity, and (5) open-mindedness. For this study, only two subscales of this instrument were used: the 6-item curiosity subscale (with a McDonald’s omega coefficient of .80) and the 5-item open-mindedness subscale (with a McDonald’s omega coefficient of .79). A 5-point Likert scale (ranging from ‘very much’ = 5 to ‘not at all’ = 1) is used to score each item. The subscale score is obtained by taking the average of the scores for each of the items of which the subscale is composed. A high score indicates a high level of curiosity or open-mindedness.

*Grit scale*. Duckworth and colleagues developed both a long version and a short version of the Grit Scale to measure perseverance and passion with respect to long-term goals [34, 36]. Both versions of this scale exhibit a 2-factor structure, including the Consistency of Interests Over Time (e.g., the reverse-scored item ‘I often set a goal but later choose to pursue a different one’) and the Perseverance of Effort (e.g., ‘I have achieved a goal that took years of work’). For this study, we used the long Spanish version of the Grit Scale [68], although we employed only the perseverance of effort subscale. The McDonald’s omega coefficient (ω) for this subscale was .70. Each item is scored on a 5-point Likert scale ranging from ‘strongly disagree’ = 1 to ‘strongly agree’ = 5.

#### Data analysis plan

Initially, outliers (values that deviate significantly from the mean) were analyzed, and then Mardia’s test was performed to evaluate multivariate skewness and kurtosis.

An exploratory factor analysis (EFA) using principal axis factoring with Oblimin rotation was conducted to study construct validity. The variance inflation factor (VIF) and tolerance were used to evaluate the collinearity of the items.

Two procedures were used to study convergent validity: (1) the average variance extracted (AVE) index indicates how well the observed variables (items) represent the latent construct (in this case, virtues). An AVE value of 0.50 or higher is considered adequate. This means that at least 50% of the variance of the indicators is explained by the latent construct, indicating good convergent validity [71]. (2) The second procedure used to study convergent validity was a correlation analysis to investigate the relationships of the new Intellectual Virtues for Interdisciplinary Research Scale (IVIRS) with other scales: the Intellectual Humility Scale [42], the perseverance of effort subscale of the Grit Scale [68], and the open-mindedness and curiosity subscales of the Virtuous Intellectual Character Scale (VICS) [51].

Moreover, the heterotrait-monotrait (HTMT) ratio of the correlations was used to test discriminant validity. “The HTMT is defined as the mean value of the item correlations across constructs relative to the (geometric) mean of the average correlations for the items measuring the same construct” [71]. HTMT values above 0.90 suggest a lack of discriminant validity for structural models with highly conceptually related constructs, and HTMT values above 0.85 suggest a lack of discriminant validity for structural models with highly conceptually unrelated constructs.

Finally, the reliability was calculated with respect to McDonald’s omega coefficient (ω) and corrected item-total correlations, which are used to assess the strength of the relationship between each item and the instrument’s total score. All these analyses were conducted via SPSS 29 software.

### Results

#### Construct validity

Initially, SPSS software was used to identify outliers for each item among the 318 cases included in the dataset. An average of 4 outlier values were found per item. Because the results did not significantly change when the outliers were removed, the outliers were retained. Moreover, Mardia’s test was performed to assess multivariate skewness and kurtosis via JASP software. The results of Mardia’s test indicated that the results were not normally distributed, with a skewness of 74.86 (p ≤ .001) and kurtosis of 594.56 (p ≤ .001).

**Fig 1:**
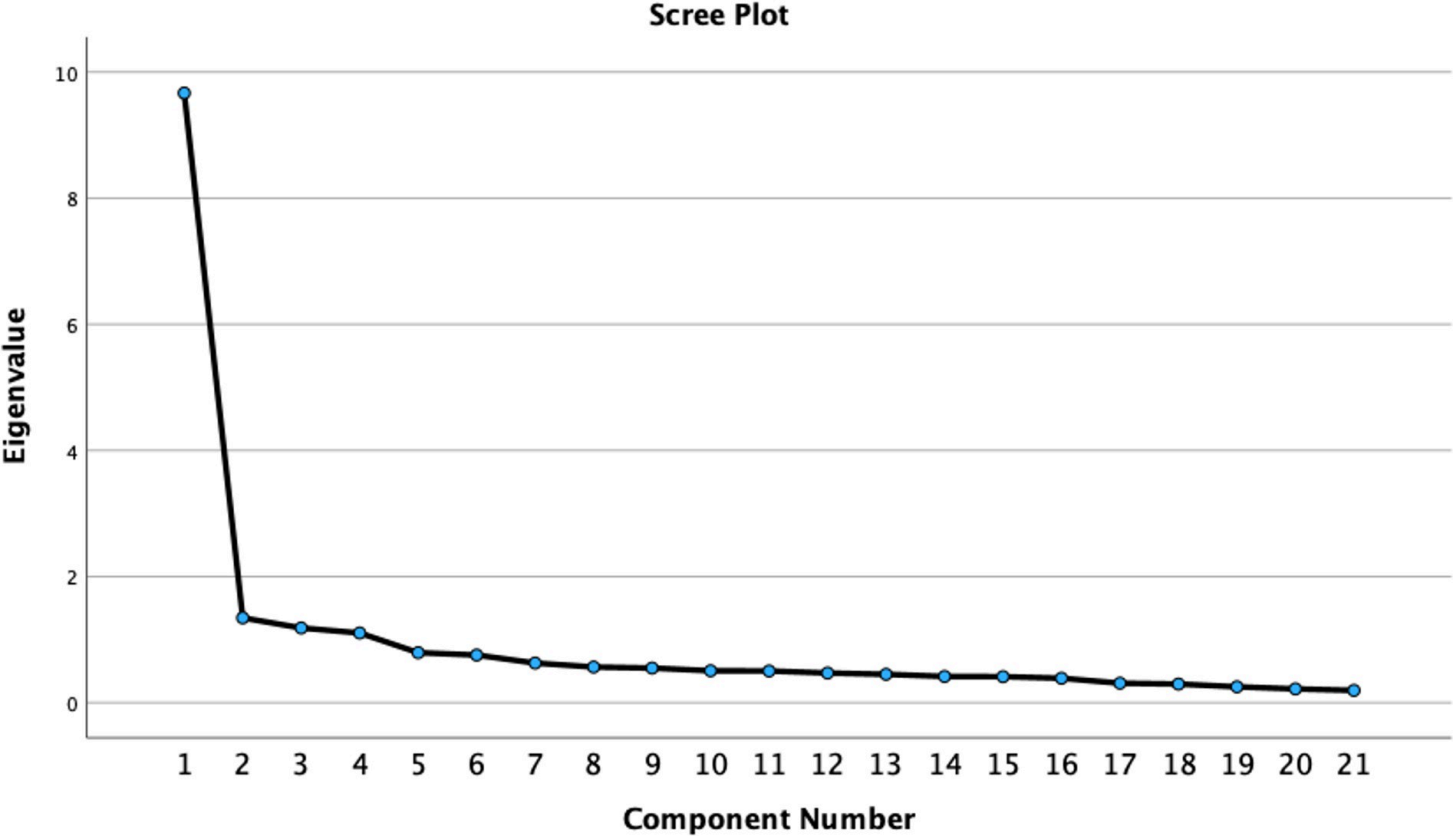
Scree plot of the IVIRS Study 1.

An exploratory factor analysis (EFA) using principal axis factoring with Oblimin rotation was conducted via SPSS. The Kaiser–Meyer–Olkin (KMO) coefficient thus obtained was .94, and Bartlett’s test of sphericity indicated a value of 3785.19, *df* = 210, *p* ≤ .001; these values suggest that the relationships among the items included in the IVIRS were sufficiently strong to proceed with the following analysis [72]. Four factors with eigenvalues greater than 1 were identified, and the scree plot also suggested four factors or dimensions (Fig 1). Moreover, following the suggestions of Worthington and Whittaker [73], we decided to preserve factors that exhibited a variance higher than 10% and were composed of at least three items. Similarly, we preserved items that exhibited a loading equal to or greater than .35.

A scale covering four dimensions and including 21 items resulted in factor loadings ranging between .35 and .89 and explained 57.63% of the total variance. Although the existence of 5 dimensions was initially postulated, exploratory analyses confirmed the existence of the following 4 dimensions: (1) intellectual empathy, (2) perseverance, (3) open-mindedness and intellectual humility, and (4) curiosity. As it was not possible to differentiate the items used to measure open-mindedness from those used to measure humility, these notions were combined into one dimension. Table 2 shows the factor loading for each item.

**Table 2:** Exploratory factor analysis using varimax rotation. Factor loading for each item (Study 1).

Original item numbering	New item numbering	Factor 1: intellectual empathy	Factor 2: open-mindedness and humility	Factor 3: perseverance	Factor 4: curiosity	Tolerance	VIF
Item 28	Item 19	.89				.34	2.95
Item 27	Item 18	.84				.35	2.85
Item 26	Item 17	.79				.40	2.52
Item 25	Item 16	.56				.49	2.06
Item 30	Item 21	.47				.65	1.53
Item 29	Item 20	.35				.52	1.91
Item 7	Item 4		.77			.57	1.76
Item 3	Item 1		.65			.58	1.73
Item 6	Item 3		.61			.53	1.87
Item 8	Item 5		.68			.43	2.30
Item 9	Item 6		.43			.64	1.57
Item 5	Item 2		.50			.53	1.90
Item 22	Item 13			.88		.35	2.87
Item 23	Item 14			.83		.30	3.29
Item 24	Item 15			.76		.29	3.50
Item 20	Item 12			.68		.47	2.14
Item 19	Item 11			.45		.46	2.17
Item 15	Item 9				.88	.32	3.10
Item 16	Item 10				.84	.32	3.16
Item 13	Item 7				.45	.43	2.31
Item 14	Item 8				.41	.49	2.04

Finally, the variance inflation factor (VIF) and tolerance were used to evaluate the collinearity of the items. Table 2 shows that all the VIF values obtained were lower than 5, and the tolerance values were greater than .20, suggesting ideal values of collinearity [71].

#### Convergent validity

The average variance extracted (AVE) index was calculated to study the convergent validity of the new instrument. The values obtained were Factor 1: intellectual empathy, AVE = .46; Factor 2: open-mindedness and humility, AVE = .38; Factor 3: perseverance, AVE = .54; and Factor 4: curiosity, AVE = .46. The factors had slightly lower AVE values compared to those suggested in the literature, with the exception of factor 3, perseverance.

Moreover, to study the convergent validity of the new instrument, a correlation analysis was conducted to explore the relationships between the IVIRS with other scales: (1) the Intellectual Humility Scale, (2) the curiosity and (3) open-mindedness subscales of the VICS, and (4) the perseverance of effort subscale of the Grit Scale. The results indicated moderate and robust associations among the total score on the IVIRS, its subscales, and the measures used to evaluate intellectual virtues (intellectual humility, curiosity, open-mindedness, and perseverance) in a general epistemic context. Table 3 shows the Pearson coefficients for all the variables included in the study.

**Table 3:** Correlations between IVIRS with intellectual virtues in general epistemic context (Study 1).

	Subscales of IVIRS				Total score IVIRS
	Intellectual empathy	Perseverance	Openness-humility	Curiosity	
Humility (Leary, et al., 2007)	.48**	.46**	.54**	.48**	.57**
Curiosity (Mesurado & Vanney, under review)	.44**	.38**	.39**	.52**	.50**
Open-mindedness (Mesurado & Vanney, under review)	.58**	.54**	.64**	.58**	.68**
Perseverance of effort (Tortul, et al., 2020)	.38**	.44**	.32**	.28**	.42**

Note

** *p*< .001

#### Discriminant validity

The heterotrait-monotrait (HTMT) ratio of the correlations was used to evaluate discriminant validity. The HTMT criterion between Factor 1, intellectual empathy and Factor 2, open-mindedness/humility, was .69; Factor 1, intellectual empathy, and Factor 3, perseverance, was .79; Factor 1, intellectual empathy and Factor 4, curiosity, was .74; Factor 3, perseverance and Factor 2, open-mindedness/humility, was .71; Factor 3, perseverance and Factor 4, curiosity, was .67; and finally, Factor 2, open-mindedness/humility and Factor 4, curiosity, was .78. No HTMT value was found above .85 or .90, suggesting the presence of discriminant validity.

#### Reliability

First, we calculated item-total correlations for all the items included in the new IVIRS. The corrected item-total correlations exceeded .51 for all the items, and the highest item-total correlation was .75. See Table 4. Second, McDonald’s omega coefficient (ω) for the total score of the scale was .94 and .87 for the intellectual empathy subscale, .89 for the perseverance subscale, .83 for the open-mindedness-humility subscale, and .86 for the curiosity subscale, thus indicating an exceptional degree of reliability.

**Table 4:** IVIRS items and corrected item—Total correlations (Study 1).

Original item numbering	New item numbering	Corrected Item-Total Correlation
Item 3	Item 1	.55
Item 5	Item 2	.60
Item 6	Item 3	.59
Item 7	Item 4	.54
Item 8	Item 5	.65
Item 9	Item 6	.54
Item 13	Item 7	.66
Item 14	Item 8	.64
Item 15	Item 9	.65
Item 16	Item 10	.65
Item 19	Item 11	.68
Item 20	Item 12	.63
Item 22	Item 13	.68
Item 23	Item 14	.72
Item 24	Item 15	.75
Item 25	Item 16	.67
Item 26	Item 17	.67
Item 27	Item 18	.69
Item 28	Item 19	.69
Item 29	Item 20	.66
Item 30	Item 21	.51

## Study 2: Confirmatory analysis of the IVIRS and its relationship with interdisciplinary research

The main objectives of Study 2 were twofold:

First, to validate the 4-factor structure of the Intellectual Virtues for Interdisciplinary Research Scale (IVIRS). In addition, we explore convergent validity via the AVE and the relationship between the intellectual virtues required to conduct interdisciplinary research and those required in a general epistemic context to provide additional evidence regarding the validity of the IVIRS. Moreover, discriminant validity and reliability were calculated.

Second, to determine whether, on the one hand, participants who had significant experience and productivity in interdisciplinary work (objective index) presented high levels of the intellectual virtues required for interdisciplinary research and, on the other hand, participants with a significant level of satisfaction regarding the results of their interdisciplinary research (subjective index) also presented high levels of these virtues.

For this second objective, the following hypotheses were formulated:

- Participants with significant experience and productivity in interdisciplinary work (objective index) and with a significant level of satisfaction regarding the results of their interdisciplinary research (subjective index) also present higher levels of the intellectual virtues required for interdisciplinary research than do participants with low levels in both indexes.
- Participants with significant experience and productivity in interdisciplinary work (objective index) and with a significant level of satisfaction regarding the results of their interdisciplinary research (subjective index) also present higher levels of the intellectual virtues required for interdisciplinary research than do participants with moderate levels in both indexes.
- Participants with moderate experience and productivity in interdisciplinary work (objective index) and with a significant level of satisfaction regarding the results of their interdisciplinary research (subjective index) also present higher levels of the intellectual virtues required for interdisciplinary research than do participants with low levels in both indexes.

### Method

#### Participants and procedure

The same data collection procedure described in Study 1 was used in Study 2. The recruitment period started on October 20, 2023, and ended on December 1, 2023. The Study 2 sample initially included 318 participants. However, 21 participants were excluded because they did not meet some of the inclusion criteria described in Study 1. The final sample included 297 academics between the ages of 24 and 80 years (mean age = 49.20, SD = 12.06); 52.2% of the participants were male, whereas 47.8% were female. In terms of the highest educational level attained by the participants, 15.5% had completed a university education, 25.3% had completed a specialization degree or a master’s degree, 39.1% had completed doctoral studies, and 20.2% had completed postdoctoral studies. Additionally, 15.2% of the participants reported performing teaching work, 26.3% reported performing disciplinary research, 15.5% reported performing interdisciplinary research, and 43.1% reported performing both types of research (disciplinary and interdisciplinary). Table 5 shows descriptive information about the participants’ interdisciplinary academic background.

**Table 5:** Descriptive information about study 2 participants’ interdisciplinary academic activity.

	0	1	2	3	4 (More than 3)
	Percentage frequency (%)	Percentage frequency (%)	Percentage frequency (%)	Percentage frequency (%)	Percentage frequency (%)
In how many interdisciplinary research projects have you participated?	16.2%	18.9%	21.9%	8.8%	34.3%
In how many conferences, workshops, etc. on interdisciplinary topics have you participated?	16.2%	10.1%	13.1%	7.4%	53.2%
How many papers have you published in the context of interdisciplinary coauthorship?	37.4%	13.1%	12.1%	6.4%	30.6%
In how many disciplines have you received systematic education?	-	23.2%	42.8%	22.6%	11.4%
How many papers have you published in journals outside your native discipline?	44.4%	11.1%	11.4%	5.1%	27.6%
How many of your publications have been cited in journals from disciplines other than your native discipline?	47.5%	12.1%	11.1%	4.7%	32.9%

#### Instruments

*Intellectual Virtues for Interdisciplinary Research Scale (IVIRS)*. The 21-item Intellectual Virtues for Interdisciplinary Research Scale (IVIRS), validated as part of Study 1, was used to measure the possession of a virtuous intellectual character regarding interdisciplinary research. The scale measures 4 factors or dimensions: (1) intellectual empathy, (2) open-mindedness and intellectual humility, (3) perseverance, and (4) curiosity. All the items are scored on a 5-point Likert scale (ranging from ‘very much’ = 5 to ‘not at all’ = 1). The score for each subscale is obtained by calculating the average of the scores attributed by the participants to the set of items of which the subscale in question is composed. The total score is obtained by calculating the average of the scores attributed by the participants to all the items. Higher scores indicate a higher level of the virtue under investigation. See S1 Appendix.

*Intellectual Humility Scale*. The 6-item scale developed by Leary and colleagues [42] was used to measure intellectual humility. The McDonald’s omega coefficient (ω) for the Intellectual Humility Scale was .76.

*Virtuous Intellectual Character Scale (VICS)*. Curiosity and open-mindedness were measured via subscales of the Virtuous Intellectual Character Scale (VICS), which was developed by Mesurado and Vanney [51] on the basis of virtue-based self-assessments [16]. The McDonald’s omega coefficients (ω) for the open-mindedness and curiosity subscales were .76 and .71, respectively.

*Grit scale*. Perseverance was measured via the Spanish version [68] of the subscale perseverance of effort of the Grit Scale, which was developed by [35, 36]. The McDonald’s omega coefficient (ω) was .71.

*Interdisciplinary Work Index*. We created an index that evaluates the participant’s level of experience and productivity in interdisciplinary research. This index includes a set of ad hoc questions (6 items) that collect *objective information* about the participant’s interdisciplinary background (see Table 5). For example, ‘In how many disciplines have you received systematic education?’ or ‘In how many interdisciplinary research projects have you participated?’. The response options included none, one, two, three, or more than three.

The six items were assigned weighting factors proportional to the duration and intensity of the interdisciplinary experience. For example, the question ‘In how many conferences, workshops, etc., on interdisciplinary topics have you participated?’ was assigned a weighting factor of 1, whereas the question ‘How many papers have you published in the context of interdisciplinary coauthorship?’ was assigned a weighting factor of 2. Similarly, the question ‘In how many disciplines have you received systematic education?’ was assigned a weighting factor of 3, the question ‘How many papers have you published in journals outside your native discipline?’ was assigned a weighting factor of 4, and the question ‘How many of your publications have been cited in journals from disciplines other than your native discipline?’ was assigned a weighting factor of 5. See S2 Appendix.

*Impact of Collaboration Scale*. The 5-item Impact of Collaboration Scale developed by Masse et al. [66] was used to evaluate the participant’s level of satisfaction regarding the results of interdisciplinary research. Three items were used to evaluate the level of satisfaction regarding *team* productivity (e.g., ‘Meeting productivity’ was measured via 6 response options, including 0 = not applicable and a range extending from 1 = very low to 5 = excellent), and two items were used to assess whether interdisciplinary research had improved the participant’s *individual* productivity and research quality (e.g., ‘Overall, interdisciplinary collaboration has improved the productivity of my research’, which was scored by reference to 6 response options, including 0 = not applicable and a range extending from 1 = strongly disagree to 5 = strongly agree). The McDonald’s omega coefficient (ω) for the Impact of Collaboration Scale was .96.

#### Data analysis plan

Initially, Mardia’s test was used to evaluate multivariate skewness and kurtosis via JASP software. To confirm the 4-factor structure of the IVIRS, a confirmatory factor analysis (CFA) was conducted with the assistance of Mplus software. The chi-square (χ^2^), ratio of χ^2^ to degrees of freedom (χ^2^/df), comparative fit index (CFI), and Tucker‒Lewis index (TLI) were used as adjustment indices for the model. Hu and Bentler [74] proposed that CFI and TLI values equal to or greater than .95 indicate good adjustment indices. Furthermore, the root mean square error of approximation (RMSEA) and standardized root mean squared residual (SRMR) were used to measure errors. Hu and Bentler [74] identified values close to .06 as indicating an acceptable level of error. The variance inflation factor (VIF) and tolerance were used to evaluate the collinearity of the items.

We planned to replicate the convergent validity study previously conducted in Study 1 with reference to a different sample. For this purpose, we performed the average variance extracted (AVE) index and correlation analysis among the IVIRS and other scales that measure open-mindedness and curiosity (two subscales of the VICS), intellectual humility, and perseverance of effort (subscale of the Grit Scale). Moreover, the heterotrait-monotrait (HTMT) ratio of the correlations was used to test discriminant validity. Additionally, we calculated the reliability with reference to McDonald’s omega coefficient (ω).

To test the hypotheses included in Study 2, the total sample was divided twice into three groups according to the results obtained by the participants (i) in the Interdisciplinary Work Index (objective index) and (ii) in the Impact of Collaboration Scale (subjective index) developed by Masse [66].

According to the Interdisciplinary Work Index, the 3 groups are: (1) participants with low levels of experience and productivity (25th percentile), (2) participants with high levels of experience and productivity (75th percentile), and (3) participants with moderate levels of experience and productivity. The three comparison groups were used because it is easier to establish a clear contrast between different levels of the variable (e.g., low, medium, and high). In turn, this facilitates the interpretation of the results and the observation of sharper patterns in performance variation between groups. Three groups provide a balance between having sufficient comparisons and maintaining clarity of results without adding unnecessary complexity. If the groups were divided into more than three, it is more likely that one of the subgroups would be too small to be compared, which may limit the validity of the statistical results. Four groups may spread participants further apart, reducing the size of each group and decreasing statistical power. In summary, dividing into three groups to compare performance allows for a clearer and more manageable analysis, while minimizing statistical noise and facilitating interpretation of the results.

One-way analysis of variance (ANOVA) was then performed using levels of experience and productivity (low, medium, and high) as independent variables and the total IVIRS score as the dependent variable. Moreover, a multivariate analysis of variance (MANOVA) was also performed using levels of experience and productivity (low, medium, and high) as independent variables and the 4 subscales of the IVIRS as dependent variables. The objective of these analyses was to determine whether individuals who presented significant levels of experience and productivity in interdisciplinary work (objective index) had also presented higher levels of the intellectual virtues pertaining to interdisciplinarity.

Finally, according to the Impact of Collaboration Scale (subjective index), the total sample was divided once again into three groups: (1) participants with low levels of satisfaction regarding the results of their interdisciplinary research (25th percentile), (2) participants with high levels of satisfaction (75th percentile), and (3) participants with moderate levels of satisfaction. One-way analysis of variance (ANOVA) and multivariate analysis of variance (MANOVA) were performed, with the level of satisfaction regarding the results of their interdisciplinary research (subjective index) as the independent variable and the total IVIRS score or the 4 subscales of the IVIRS score as the dependent variable.

To perform these analyses, we used SPSS 29 software and conducted ANOVA, MANOVA, correlation, and McDonald’s omega coefficient (ω) analyses.

### Results

#### Confirmatory factor analysis

Study 2 aimed to confirm the 4-factor structure of the IVIRS found in Study 1. Initially, Mardia’s test was performed to assess multivariate skewness and kurtosis. The results of Mardia’s test indicated that the results were not normally distributed, with a skewness of 72.74 (*p ≤* .001) and kurtosis of 584.85 (*p ≤* .001).

**Fig 2:**
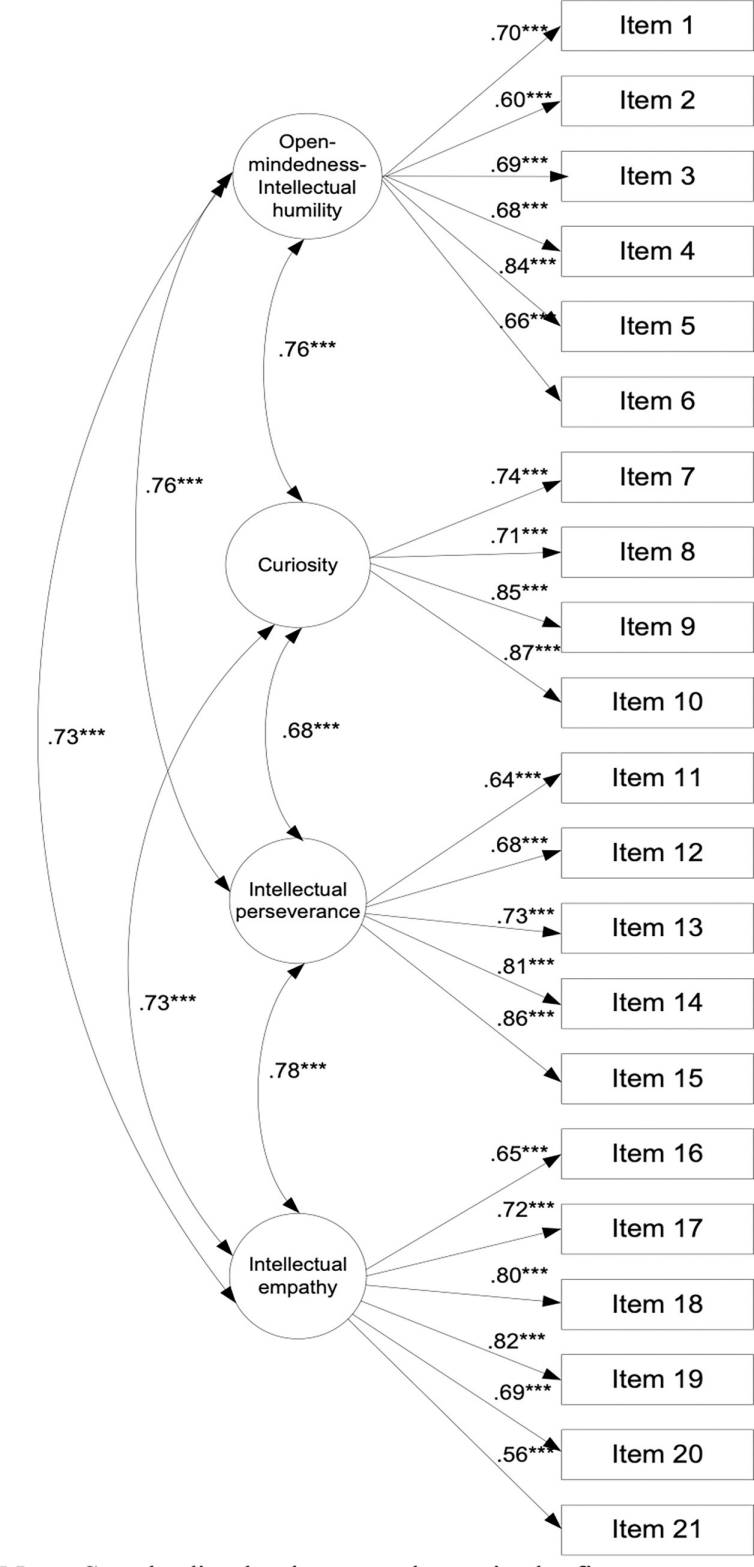
The path diagram of the IVIRS for the Study 2.

A confirmatory factor analysis using maximum likelihood parameter estimates with standard errors and a mean-adjusted chi-square test statistic, which is robust to nonnormality, was performed to test the theoretical model, which indicated that the model exhibited an excellent fit, *χ*^*2*^ = 273.70, df = 183, *p* ≤. 001, *χ*^*2*^*/df* = 3.41, CFI = .97, TLI = .96, SRMR = .04, RMSEA = .04 (90% CI: 03-.05). Fig 2 presents the estimated value alongside the significance level for each item included in the new scale.

Moreover, the variance inflation factor (VIF) and tolerance were used to evaluate the collinearity of the items. Table 5 shows that all the VIF values obtained with the second participant sample were also lower than 5, and the tolerance values were greater than .20, suggesting ideal values of collinearity [71, 75]. See Table 6.

**Table 6:** Multicollinearity test on the second sample of each item of VICS (Study 2).

Original item numbering	New item numbering	Tolerance	VIF
Item 28	Item 19	.36	2.79
Item 27	Item 18	.40	2.48
Item 26	Item 17	.47	2.14
Item 25	Item 16	.55	1.82
Item 30	Item 21	.67	1.49
Item 29	Item 20	.52	1.93
Item 22	Item 13	.52	1.91
Item 23	Item 14	.39	2.58
Item 24	Item 15	.33	3.07
Item 20	Item 12	.52	1.91
Item 19	Item 11	.52	1.93
Item 7	Item 4	.54	1.87
Item 3	Item 1	.54	1.85
Item 6	Item 3	.54	1.87
Item 8	Item 5	.38	2.66
Item 9	Item 6	.56	1.79
Item 5	Item 2	.60	1.67
Item 15	Item 9	.34	2.96
Item 16	Item 10	.31	3.28
Item 13	Item 7	.46	2.19
Item 14	Item 8	.51	1.95

#### Convergent validity

The average variance extracted (AVE) index was calculated to study the convergent validity of the new instrument. The values obtained were Factor 1: intellectual empathy, AVE = .51; Factor 2: perseverance, AVE = .56; Factor 3: open-mindedness and humility, AVE = .49; and Factor 4: curiosity, AVE = .63. All the factors obtained good AVE values except for the open-mindedness‒humility factor, which had slightly lower AVE values than those suggested by the literature.

Moreover, to confirm the convergent validity of the IVIRS with respect to a new and independent sample, a correlation analysis was conducted with respect to the new IVIRS and other scales: (1) the Intellectual Humility Scale, (2) the curiosity subscale and (3) the open-mindedness subscale of the VICS, and (4) the perseverance subscale of the Grit Scale. As in Study 1, the results indicated moderate and robust associations among the total score of the IVIRS, its subscales, and other scales that measure intellectual virtues (intellectual humility, curiosity, open-mindedness, and perseverance) in a general epistemic context. Table 7 shows the Pearson coefficients for all the variables included in the study.

**Table 7:** Correlations between IVIRS with Intellectual Virtues in a general epistemic context (Study 2).

	Subscales of IVIRS				Total score IVIRS
	Intellectual empathy	Perseverance	Openness-humility	Curiosity	
Humility (Leary, et al., 2007)	.42**	.35**	.52**	.44**	.50**
Curiosity (Mesurado & Vanney, under review)	.48**	.40**	.44**	.54**	.54**
Open-mindedness (Mesurado & Vanney, under review)	.56**	.45**	.56**	.47**	.59**
Perseverance of effort (Tortul, et al., 2020)	.28**	.37**	.32**	.22**	.35**

Note

** *p*< .001

#### Discriminant validity

The heterotrait-monotrait (HTMT) ratio of the correlations was used to test discriminant validity. The HTMT criterion between Factor 1, intellectual empathy, and Factor 2, perseverance, was .79; Factor 1, intellectual empathy, and Factor 3, open-mindedness/humility, was .75; Factor 1, intellectual empathy, and Factor 4, curiosity, was .75; Factor 2, perseverance, and Factor 3, open-mindedness/humility, was .76; Factor 2, perseverance, and Factor 4, curiosity, was .73; and lastly, Factor 3, open-mindedness/humility, and Factor 4, curiosity, was .80. No HTMT value was found above .85 or .90, suggesting the presence of discriminant validity.

#### Reliability

Finally, in Study 2, the McDonald’s omega coefficient (ω) for the total score of the scale was .94, and the corresponding coefficients were .85 for the intellectual empathy subscale, .86 for the perseverance subscale, .84 for the open-mindedness and intellectual humility subscale, and .88 for the curiosity subscale, which indicate the exceptional reliability of the scale.

#### Levels of experience and productivity in the context of interdisciplinary work (objective index) and the IVIRS

The second objective of Study 2 was to determine whether individuals with significant experience and productivity in interdisciplinary work (objective index) also present high levels of the intellectual virtues required for interdisciplinary research. As it was mentioned previously, to achieve this goal, the total sample was split into three groups (low, moderate or high levels of experience and productivity according to the Interdisciplinary Work Index). The frequency with which the participants were included in different groups and the mean and standard deviation of the level of intellectual virtues for interdisciplinary research for each group are presented in Table 8.

**Table 8:** Mean and standard deviation and Bonferroni post hoc test of IVIRS between participants with high, middle, and low levels of experience and productivity in interdisciplinary work for Study 2.

	High level n = 82		Middle level n = 137		Low level n = 78		Bonferroni test (95% confidence interval)		
	M	SD	M	SD	M	SD	High vs Low	High vs Middle	Middle vs Low
Total Score IVIRS	4.35	.42	4.09	.50	3.84	.52	.51** (.33, .70)	.26** (.10, .43)	.25** (.08, .41)
Openness-Humility	4.44	.48	4.23	.51	3.99	.58	.44** (.25, .64)	.21** (.03, .39)	.23** (.06, .41)
Intellectual empathy	4.16	.53	3.96	.55	3.78	.52	.38** (.17, .58)	.19* (.01, .38)	.18* (.00, .36)
Perseverance	4.31	.51	3.93	.64	3.64	.65	.68** (.44, .91)	.38** (.18, .59)	.29** (.08, .50)
Curiosity	4.51	.49	4.24	.67	3.95	.68	.55** (.31, .79)	.27** (.05, .48)	.28** (.07, .50)

Note

** *p*< .001

* *p*< .05

The results indicated that participants with significant levels of experience and productivity in the context of interdisciplinary work also presented high levels of the intellectual virtues required for interdisciplinary research [*F* (2, 296) = 22.13, *p* <. 001, *eta* = .20 (95% CI = .06, .20)]. The Bonferroni post hoc test indicated that participants with significant levels of experience and productivity in interdisciplinarity also presented higher levels of the intellectual virtues required for interdisciplinary research than did participants who had moderate (β = .26, 95% CI = .10, .43) or low levels (β = .51, 95% CI = .33, .70) of experience and productivity. In addition, participants with moderate levels of experience and productivity in interdisciplinary work presented higher levels of these intellectual virtues than did participants with low levels of experience and productivity (β = .25, 95% CI = .08, .41).

A MANOVA was subsequently performed using levels of experience and productivity (high, medium, and low) as independent variables and the 4 subscales of the IVIRS as dependent variables. The goal of this analysis was to investigate whether the general differences between the level of experience and productivity in interdisciplinarity and the level of the intellectual virtues required for interdisciplinary research were maintained when each dimension of the IVIRS was analyzed separately. The results indicated significant differences in MANOVA Hotelling’s Trace F (8, 580) = 8.41, *p <* .001, *eta* = .09. Moreover, differences were found among the 4 factors of the IVIRS: intellectual empathy [*F* (2, 294) = 9.68, *p* <. 001, *eta* = .06], perseverance [*F* (2, 294) = 24.59, *p* <. 001, *eta* = .14], open-mindedness-humility [*F* (2, 294) = 14.56, *p* <. 001, *eta* = .09], and curiosity [*F* (2, 294) = 15.37, *p* < .001, *eta* = .10].

The Bonferroni post hoc test indicated that participants with high levels of experience and productivity in interdisciplinary research also exhibited higher levels of open-mindedness and intellectual humility, intellectual empathy, perseverance, and curiosity than did participants with moderate or low levels of experience and productivity. In addition, participants with moderate levels of experience and productivity presented higher levels of open-mindedness and intellectual humility, intellectual empathy, perseverance, and curiosity than did participants with low levels of experience and productivity. See Table 8.

#### Level of satisfaction regarding the results of interdisciplinary research (subjective index) and the IVIRS

A one-way analysis of variance (ANOVA) was conducted to determine whether participants who had a significant level of satisfaction regarding the results of their interdisciplinary research (subjective index) also presented high levels of the intellectual virtues required for interdisciplinary research.

The results indicated that participants with significant levels of satisfaction also presented high levels of the intellectual virtues [*F* (2, 296) = 30.56, *p* <. 001, *eta* = .17 (95% CI = .10, .25)]. The Bonferroni post hoc test indicated that participants with significant levels of satisfaction also presented higher levels of the intellectual virtues than did participants who had moderate (β = .30, 95% CI = .14, .47) or low levels (β = .59, 95% CI = .41, .78) of satisfaction. In addition, participants with moderate levels of satisfaction presented higher levels of the intellectual virtues than did participants with low levels of satisfaction (β = .29, 95% CI = .13, .45).

A MANOVA was subsequently performed using the level of satisfaction (high, medium, and low) as independent variable and the 4 subscales of the IVIRS as dependent variables. The goal of this analysis was to investigate whether the general differences between the level of satisfaction and the level of each intellectual virtue were maintained when each dimension of the IVIRS was analyzed separately. The results indicated significant differences in MANOVA Hotelling’s Trace F (8, 580) = 8.30, *p <* .001, *eta* = .10. Moreover, differences were found among the 4 factors of the IVIRS: intellectual empathy [*F* (2, 294) = 18.87, *p* <. 001, *eta* = .11], perseverance [*F* (2, 294) = 27.40, *p* <. 001, *eta* = .16], open-mindedness-humility [*F* (2, 294) = 22.23, *p* <. 001, *eta* = .13], and curiosity [*F* (2, 294) = 18.53, *p* <. 001, *eta* = .11].

The Bonferroni post hoc test indicated that participants with significant levels of satisfaction also presented higher levels of open-mindedness and intellectual humility, intellectual empathy, perseverance, and curiosity than did participants with moderate or low levels of satisfaction. Additionally, participants with moderate levels of satisfaction presented higher levels of open-mindedness, intellectual humility, intellectual empathy, perseverance, and curiosity than did participants with low levels of satisfaction. See Table 9.

**Table 9:** Mean and standard deviation and Bonferroni post hoc test of IVIRS between participants with high, middle, and low levels of satisfaction regarding the results of interdisciplinary research for Study 2.

	High level n = 78		Middle level n = 140		Low level n = 79		Bonferroni test (95% confidence interval)		
	M	SD	M	SD	M	SD	High vs Low	High vs Middle	Middle vs Low
Total Score IVIRS	4.39	.43	4.09	.45	3.80	.55	.59** (.41, .78)	.30** (.14, .47)	.29** (.13, .45)
Openness-Humility	4.51	.47	4.20	.45	3.97	.63	.54** (.34, .74)	.30** (.13, .48)	.24** (.06, .41)
Intellectual empathy	4.23	.53	3.95	.52	3.72	.52	.51** (.31, .71)	.28** (.10, .46)	.24** (.06, .41)
Perseverance	4.32	.57	3.93	.59	3.61	.67	.72** (.48, .95)	.39** (.19, .60)	.32** (.12, .53)
Curiosity	4.51	.54	4.26	.60	3.91	.73	.60** (.36, .84)	.25** (.03, .46)	.36** (.14, .57)

Note

** *p*< .001

* *p*< .05

## Discussion

This research makes a significant contribution by designing and validating a psychometric tool to assess crucial intellectual virtues that researchers must possess for interdisciplinarity. The proposal’s novelty lies in conducting an empirical investigation on the basis of the theoretical framework of virtue epistemology, thus bridging the gap between empirical psychology and philosophy. Although there are several scales for assessing some intellectual virtues individually, very few instruments evaluate intellectual character comprehensively, i.e., including several epistemic virtues in the same construct. Moreover, assessing the specific features of interdisciplinary researchers’ intellectual character offers groundbreaking insights as they provide an empirical approach to evaluating the relationship dynamics between the challenges posed by interdisciplinarity and some epistemic virtues, understood as traits of a virtuous intellectual character. The scale is valuable because it can be a key initial step in promoting interdisciplinarity by focusing on developing specific qualities in researchers.

On the basis of the results obtained by Vanney and colleagues [20], who carried out a qualitative study to identify the intellectual virtues that scholars should possess to undertake successful interdisciplinary projects, we initially chose five intellectual virtues as part of our new scale: (1) intellectual humility, (2) open-mindedness, (3) intellectual perseverance, (4) curiosity, and (5) intellectual empathy.

However, the EFA conducted for Study 1 identified only four dimensions, as the pools of items that were initially developed to measure intellectual humility and open-mindedness converged into a unique factor. In turn, the confirmatory factor analysis conducted for Study 2 corroborated the four-dimensional structure observed in Study 1. Therefore, the new instrument has four conceptually and empirically differentiated dimensions.

First, the "*intellectual empathy*" dimension was the most decisive factor on the scale. The findings suggest that intellectual empathy is crucial for comprehending the cognitive process of researchers from other fields. Indeed, interdisciplinary work requires understanding how other researchers think, their methodological approach, and how they arrive at their conclusions. It is necessary to adopt the way in which other researchers approach a question and follow their reasoning methods.

The "*open-mindedness and intellectual humility*" was the second relevant dimension. Successful interdisciplinary research requires being open-minded to different perspectives, taking into account findings from various fields, being willing to adjust one’s beliefs when necessary, trusting the expertise of others, accepting criticism from others, and acknowledging one’s own mistakes.

The third factor was "*intellectual perseverance*." To achieve success in interdisciplinarity, one must reach the end goal through arduous work, strive despite encountering difficulties, avoid becoming discouraged, and find ways of overcoming challenges. It is essential to persist until one has obtained some understanding of the language, methodology, and perspectives of the other disciplines involved in the research.

Lastly, "*curiosity*" was the fourth dimension. Curiosity concerning the problems and approaches associated with disciplines other than one’s own could represent a fundamental prerequisite for beginning an interdisciplinary inquiry as well as a motivation for sustaining work over time. Interdisciplinary research involves curiosity about how different disciplines approach the topic under study, enthusiasm concerning the contributions of various fields, and interest in their explanations. Furthermore, interdisciplinarity requires researchers to value discussions with scholars from other areas as a stimulus for new questions that can, in turn, lead to new ideas.

Our results revealed an overlap between intellectual humility and open-mindedness, indicating that these virtues are not empirically distinguished in IVIRS. Although these two epistemic virtues have been distinguished theoretically [76] and empirically [42], the differences between them are subtle. At its metacognitive core, intellectual humility involves recognizing the limits of one’s own knowledge and being aware of one’s own fallibility. On the other hand, open-mindedness involves fairly or impartially considering diverse perspectives, which does not necessarily require the assumption of the limitations of one’s own beliefs. However, in an interdisciplinary research context, these two virtues act in conjunction and reinforce each other. In fact, some IVIRS items incorporate elements of both. For example, in item 1 (originally intellectual humility), "When researching, I am willing to acknowledge mistakes based on evidence provided by other disciplines," the humble attitude implies a prior openness to evidence from other disciplines. Likewise, in item 6 (originally open-mindedness), "When researching, I am open to considering results from other disciplines even if they are not consistent with those achieved by my field,” the open-minded attitude assumes a prior humble recognition that the knowledge of one’s own discipline is not absolute.

In addition, Studies 1 and 2 also analyzed the associations of the IVIRS with other scales that measure intellectual virtues (open-mindedness, curiosity, intellectual humility, and perseverance) in a general epistemic context. For example, the Virtuous Intellectual Character Scale [51] assesses open-mindedness with the item ‘I am open to considering new evidence’ that may refer to the same discipline. In contrast, the IVIRS item ‘When researching, I am open to taking into account perspectives from other disciplines’ requires an open disposition also about other fields. Concerning intellectual humility, in the scale developed by Leary [42], the item ‘I accept that my beliefs and attitudes may be wrong’ may apply to the disciplinary domain, while the IVIRS item ‘When I do research, I am willing to acknowledge mistakes based on evidence from colleagues in other disciplines’ demands a deeper humble attitude that includes other disciplines as well. The same applies to perseverance. Assessing tenacity, the item ‘I have overcome setbacks to conquer an important challenge’ from the Grit Scale [36] may apply within a disciplinary context, while the IVIRS item ‘When researching, I look for ways to overcome the difficulties caused by interdisciplinarity’ makes perseverance explicitly refer to interdisciplinarity.

The results indicate moderate and robust associations between the IVIRS and different scales that measure virtues in a general epistemic context, providing further evidence to support the validity of the new scale with reference to two different samples. Importantly, as can be seen in Tables 3 and 7, in most cases, the levels of association between intellectual virtues in the interdisciplinary context (measured with IVIRS) and the same virtue assessed in a general epistemic context (measured with other instruments) are stronger correlations. In contrast, the level of association between different virtues tends to be slightly lower. For example, Table 7 shows that the level of correlation between the assessment of curiosity (measured with IVIRS and with VICS) is .54, while the levels of association between curiosity (measured with IVIRS) and other intellectual virtues (humility, open-mindedness, and perseverance of effort) measured with other instruments drop to .22-.47. In both Studies 1 and 2, the correlation levels obtained are moderate indicating no overlap between the measures. That is, in the IVIRS measurements, the characteristics of the virtues are preserved, although they make explicit reference to the interdisciplinary context. Moreover, the HTMT ratio of the correlations confirmed the discriminant validity, indicating no overlap between the items within the factor. That is, each of the items provides information about a different aspect of a virtue.

Furthermore, Study 2 also demonstrated that researchers with considerable expertise, productivity, and satisfaction in the context of interdisciplinary investigation (measured via objective and subjective indices) presented elevated levels of the intellectual virtues previously identified. This finding was evident both in their overall scores on the assessment of intellectual virtues—i.e., researcher intellectual character—and in the scores associated with each of the virtues individually—i.e., open-mindedness and intellectual humility, intellectual empathy, intellectual perseverance, and curiosity. In contrast, the study also revealed that researchers who had (i) only limited interdisciplinary experience and productivity (objective index) and (ii) a low level of satisfaction regarding the results of their interdisciplinary collaboration (subjective index) presented lower levels of the virtues analyzed. Once again, this finding was reflected in both the overall and the individual scores for each virtue. The results of Study 2 are consistent with theoretical proposals previously developed in virtue epistemology [31, 39, 40, 77, 78].

In summary, both Study 1 and Study 2 provided strong evidence to support the internal consistency of the IVIRS, which is a reliable scale whether the overall score is used or whether each of its four dimensions or subscales are utilized separately, i.e., (i) open-mindedness and intellectual humility, (ii) intellectual empathy, (iii) intellectual perseverance, and (iv) curiosity.

### Limitations

The participants in the studies reported in this paper were researchers from Latin America, which limits the generalizability of the conclusions. Although contemporary research is highly globalized, sociocultural characteristics may have influenced the results of these studies. For example, it is possible that the relevance of intellectual empathy is greater in cultures in which warm interpersonal relationships are highly valued, such as Latin American cultures. It would be desirable for this research to inspire follow-up studies that can test the generalizability of the results with reference to populations that were not analyzed in this investigation.

According to the literature [79], the invariance analysis is important since it allows us to demonstrate that a measurement could be replicated in groups with different characteristics. In future studies, it would be interesting to consider gender, research areas, years of experience in interdisciplinarity, and cultural contexts to study the structural and measurement invariance of the new scale.

## Conclusions and directions for future research

The investigation presented in this article elucidates the intellectual character of interdisciplinary researchers by analyzing the intellectual virtues that they must possess to work effectively alongside colleagues from diverse fields. Some character traits are found to be essential and crucial with respect to increasing the productivity and impact of collaboration.

On the one hand, this research highlights new opportunities to foster collaborative inquiry in fields that, by their very nature, are interdisciplinary, such as cognitive science, climate change, or science and religion. To date, the most significant efforts in these fields have focused on promoting and consolidating organizational structures that can facilitate interdisciplinarity, such as adequate infrastructure, community building, institutional policies, and funding. However, although all these efforts are necessary, the results of this research indicate that further effort is needed. Perhaps the time has come to move in a new direction by focusing more closely on the development of people’s qualities than on the capacities of institutions since, after all, people, not disciplines, are the actors who engage in dialog.

On the other hand, this paper also paves the way for future studies that seek to enhance the intellectual character of interdisciplinary researchers, for example, by developing interventions and training programs. To analyze the effectiveness of such initiatives, instruments such as the one designed and validated here will be indispensable.

## Supporting information

S1 Appendix Intellectual Virtues for Interdisciplinary Research Scale (IVIRS). (DOCX)

S2 Appendix Interdisciplinary Work Index. (DOCX)
