# Supplementary material for: Measuring qualities needed for interdisciplinary work: The Intellectual Virtues for Interdisciplinary Research Scale (IVIRS)
Source: PLoS One. 2024 Nov 15;19(11):e0312938. doi: 10.1371/journal.pone.0312938 (PMC11567625; doi:10.1371/journal.pone.0312938)
Supplement: S1 Appendix — (DOCX) [file pone.0312938.s001.docx]

**S1 Appendix. Intellectual Virtues for Interdisciplinary Research Scale (IVIRS)**

A 5-level Likert scale (‘very much’ = 5 to ‘not at all’ =1) is used to measure each item.

|  | **Items in Spanish** | **Items in English** | **Dimension** |
| --- | --- | --- | --- |
| 1 | Cuando investigo, estoy dispuesto a reconocer errores a partir de evidencias aportadas por otras disciplinas. | When researching, I am willing to acknowledge mistakes based on evidence provided by colleagues from other disciplines | Open-mindedness and humility |
| 2 | Cuando investigo, me apoyo en el conocimiento de expertos de otras disciplinas. | When researching, I rely on the knowledge of experts from other disciplines. | Open-mindedness and humility |
| 3 | Cuando investigo, acepto que la crítica de colegas de otras áreas pueden mejorar mi trabajo. | When researching, I accept that criticism from colleagues in other areas can improve my work. | Open-mindedness and humility |
| 4 | Cuando investigo, estoy dispuesto a cambiar lo que pienso luego de un intercambio con colegas de otras disciplinas. | When researching, I am willing to change what I think after an exchange with colleagues from other disciplines. | Open-mindedness and humility |
| 5 | Cuando investigo, estoy abierto a tener en cuenta perspectivas de otras disciplinas. | When researching, I am open to taking into account perspectives from other disciplines. | Open-mindedness and humility |
| 6 | Cuando investigo, estoy abierto a considerar resultados de otras disciplinas aunque no sean consistentes con los alcanzados por mi campo. | When researching, I am open to consider results from other disciplines even if they are not consistent with those achieved by my field. | Open-mindedness and humility |
| 7 | Cuando investigo, me entusiasma conocer aportes de otras disciplinas. | When researching, I get excited about knowing the contributions of other disciplines. | Curiosity |
| 8 | Cuando investigo, las discusiones con investigadores de disciplinas diferentes me suscitan nuevas preguntas. | When researching, discussions with researchers from different disciplines raise new questions for me. | Curiosity |
| 9 | Cuando investigo, me gusta preguntarme cómo se aborda un tema desde otras disciplinas. | When researching, I like to ask myself how a topic is approached from other disciplines. | Curiosity |
| 10 | Cuando investigo, me da curiosidad conocer cómo se explican las cosas desde disciplinas diferentes. | When researching, I am curious to learn how things are explained in different disciplines. | Curiosity |
| 11 | Cuando investigo, soy constante hasta comprender adecuadamente el lenguaje de las otras disciplinas. | When researching, I am constant until I understand the language of other disciplines adequately. | Perseverance |
| 12 | Cuando investigo, las dificultades de la interdisciplinariedad no me desaniman. | When researching, the difficulties of interdisciplinarity do not discourage me. | Perseverance |
| 13 | Cuando investigo, me esfuerzo por llegar hasta el final aunque el trabajo interdisciplinar sea arduo. | When researching, I strive to reach the end goal even though interdisciplinary work is arduous. | Perseverance |
| 14 | Cuando investigo, estoy dispuesto a dedicar el tiempo necesario para entender un tema interdisciplinarmente. | When researching, I am willing to devote the time necessary to understand a subject in an interdisciplinary way. | Perseverance |
| 15 | Cuando investigo, busco el modo de superar las dificultades ocasionadas por la interdisciplinariedad. | When researching, I look for ways to overcome the difficulties caused by interdisciplinarity. | Perseverance |
| 16 | Cuando trabajo con personas de otras áreas, puedo adoptar sus modos de estudiar la cuestión. | When working with people from other fields, I am able to adopt their ways of studying the issue. | Intellectual empathy |
| 17 | Cuando trabajo con personas de otras áreas, trato de comprender cómo están pensando el problema los demás. | When working with people from other fields, I try to understand how others are thinking about the problem. | Intellectual empathy |
| 18 | Cuando trabajo con personas de otras áreas, busco entender el abordaje metodológico de los otros investigadores. | When working with people from other fields, I seek to understand the methodological approach of other researchers. | Intellectual empathy |
| 19 | Cuando trabajo con personas de otras áreas, trato de entender de qué manera ellos llegan a determinadas conclusiones. | When working with people from other fields, I try to understand how they reach certain conclusions. | Intellectual empathy |
| 20 | Cuando trabajo con personas de otras áreas, intento adoptar sus perspectivas para enriquecer mi conocimiento con abordajes diversos. | When working with people from other fields, I try to adopt their perspectives to enrich my knowledge with diverse approaches. | Intellectual empathy |
| 21 | Cuando trabajo con personas de otras áreas, puedo seguir sus procesos de razonamiento. | When working with people from other fields, I am able to follow their reasoning processes. | Intellectual empathy |
