# Supplementary material for: Measuring qualities needed for interdisciplinary work: The Intellectual Virtues for Interdisciplinary Research Scale (IVIRS)
Source: PLoS One. 2024 Nov 15;19(11):e0312938. doi: 10.1371/journal.pone.0312938 (PMC11567625; doi:10.1371/journal.pone.0312938)
Supplement: S2 Appendix — (DOCX) [file pone.0312938.s002.docx]

**S2 Appendix. Interdisciplinary Work Index**

| Answer using the following scale: 0, 1, 2, 3, 4 (more than three). | | | |
| --- | --- | --- | --- |
|  |  |  |  |
|  | **Items in Spanish** | **Items in English** | **Weighting factor** |
| 1 | ¿En cuántos proyectos de investigación interdisciplinar ha participado? | In how many interdisciplinary research projects have you participated? | 1 |
| 2 | ¿En cuántos congresos, workshops, etc., de temáticas interdisciplinares ha participado? | In how many conferences, workshops, etc., on interdisciplinary topics have you participated? | 1 |
| 3 | ¿Cuántos trabajos ha publicado en coautoría interdisciplinar? | How many papers have you published in the context of interdisciplinary coauthorship? | 2 |
| 4 | ¿En cuantas disciplinas ha recibido educación sistemática? | In how many disciplines have you received systematic education? | 3 |
| 5 | ¿Cuántos trabajos ha publicado en revistas fuera de su disciplina nativa? | How many papers have you published in journals outside your native discipline? | 4 |
| 6 | ¿Cuántas de sus publicaciones han sido citadas en revistas de disciplinas distintas a su disciplina nativa? | How many of your publications have been cited in journals from disciplines other than your native discipline? | 5 |
|  |  |  |  |
